# Research on the Impact of Workaholic Leadership on Newcomer Organizational Socialization

**DOI:** 10.3390/bs16081292

**Published:** 2026-07-29

**Authors:** Hui Deng, Jinmei Wu, Xian Cheng

**Affiliations:** School of Economics and Management, Beijing Forestry University, Beijing 100083, China; hdeng0220@bjfu.edu.cn (H.D.);

**Keywords:** workaholic leadership, work engagement, leader identification, newcomer organizational socialization

## Abstract

To investigate the mechanisms underlying how workaholic leadership influences newcomers’ organizational socialization outcomes, this study integrates social learning theory to construct a moderated mediation model, introducing newcomer work engagement as the mediator and leader identification as the moderator. Utilizing a sample of 375 newcomers from a textile and apparel enterprise in Shandong Province, China, our empirical analysis revealed that workaholic leadership fosters newcomers’ task mastery, role clarity, and social integration by enhancing their work engagement. Furthermore, leader identification not only amplified the positive effect of workaholic leadership on newcomer work engagement but also strengthened its indirect effect via work engagement. These findings extend the literature on newcomer socialization and leadership dynamics, offering both theoretical advancements and actionable insights for managers to navigate workaholic leadership and facilitate newcomers to integrate into the organization.

## 1. Introduction

As business competition intensifies and work environments become more dynamic, helping newcomers adjust successfully has grown into a key concern for both managers and scholars. Accordingly, organizational socialization has attracted sustained research attention. It refers to the process through which employees with less than one year of organizational tenure, defined as newcomers (Bauer et al., 2025), adjust to their work environment and fit into their teams and organizations. This process encompasses both how newcomers acquire the knowledge and skills necessary to adapt to their roles and how the organization influences them while interacting with their self-regulation (Chow, 2002). Chao et al. (1994) proposed three key indicators of successful socialization outcomes: task mastery, role clarity, and social integration, which separately reflect how well newcomers adapt to job tasks, understand role requirements, and build interpersonal connections at work. Prior studies have confirmed the significance of successful socialization for both newcomers and organizations. For newcomers, it reduces turnover intentions, improves organizational commitment, and enhances job performance (Chong et al., 2021; A. S. Lee & Jacobs, 2024). For organizations, it also helps sustain a consistent organizational culture and improve overall operational performance (Saks & Gruman, 2014).

Scholars have identified a wide range of antecedents that predict newcomer socialization, including organizational socialization tactics (Cai et al., 2023), leadership styles (Nifadkar, 2020), mentoring and coworker support (Gardner et al., 2022), as well as newcomers’ personal traits (Frögéli et al., 2023). Among these factors, leadership stands out as a critical situational predictor of leaders’ daily interactions with newcomers. Most existing research has focused on supportive leadership styles, such as inclusive leadership, authentic leadership (Calderon-Mafud & Pando-Moreno, 2018), transformational leadership (L. Y. Lee et al., 2013), and servant leadership (Bauer et al., 2019). Overall, these studies converge that supportive leaders facilitate newcomer adjustment via explicit support, role clarification and work empowerment. However, workaholic leadership operates in a fundamentally different way. Unlike supportive leaders who actively help newcomers adapt, workaholic leaders seldom provide targeted developmental assistance. Instead, they influence newcomers indirectly by conveying implicit work norms through their own excessive work behaviors. Although workaholic leadership is widely observed in practice, it has been largely overlooked in existing socialization research.

Workaholic leadership is highly prevalent in Chinese organizational settings due to the traditional Confucian culture that advocates hard work, dedication and perseverance, and growing workplace competition across industries (Y. Xu & Li, 2021). Workaholic leadership refers to leaders who are emotionally enthusiastic, cognitively preoccupied, and behaviorally invest substantial time and energy in work, ruminating on work issues even during off-hours, with most of their cognition, emotion and daily behaviors revolving around work (Schaufeli et al., 2008). Prior empirical studies have explored mixed outcomes associated with workaholic leadership. On the positive side, such leaders can motivate employee self-presentation (Zeng & Liu, 2022), stimulate individual creativity (She et al., 2024), and improve team and firm-level performance (She et al., 2021). Nonetheless, prevailing scholarship has predominantly highlighted its dark side. The relentless high work expectations and implicit work pressure conveyed by workaholic leaders have been verified to trigger employee emotional exhaustion (Zhan et al., 2026) and chronic work fatigue (Qiu et al., 2025). Despite these existing findings, most relevant studies target experienced employees, while overlooking how workaholic leadership functions among newcomers during their socialization process.

Newcomers differ substantially from experienced employees, especially in the Chinese cultural context. Chinese collectivist culture highlights leaders’ role modeling and top-down behavioral influence, making them important carriers of workplace norms (X. P. Chen et al., 2014). In this context, employees value collective interests and interpersonal harmony and tend to follow behavioral expectations conveyed by leaders and organizations (Den Hartog & De Hoogh, 2024; Fan & Wanous, 2008). Compared with experienced employees who have already adapted to organizational rules and role demands, newcomers lack work experience and a clear understanding of their roles in the early stages of entry (Peltokorpi et al., 2022). Therefore, they primarily rely on leaders’ behavioral cues to understand implicit workplace norms and adjust their own behaviors to fit organizational requirements (Bauer et al., 2007). When leaders demonstrate high work dedication, newcomers typically view such behaviors as organizational work standards and voluntarily increase their work engagement to facilitate role adaptation and organizational integration (Zeng & Liu, 2022). Accordingly, although workaholic leadership generally generates negative outcomes for employees through intense work demands, its strong work-oriented behavioral demonstration may facilitate newcomers’ socialization. Nevertheless, empirical research has yet to examine this potential positive effect, leaving an important research gap in the organizational socialization literature.

**Figure 1:**
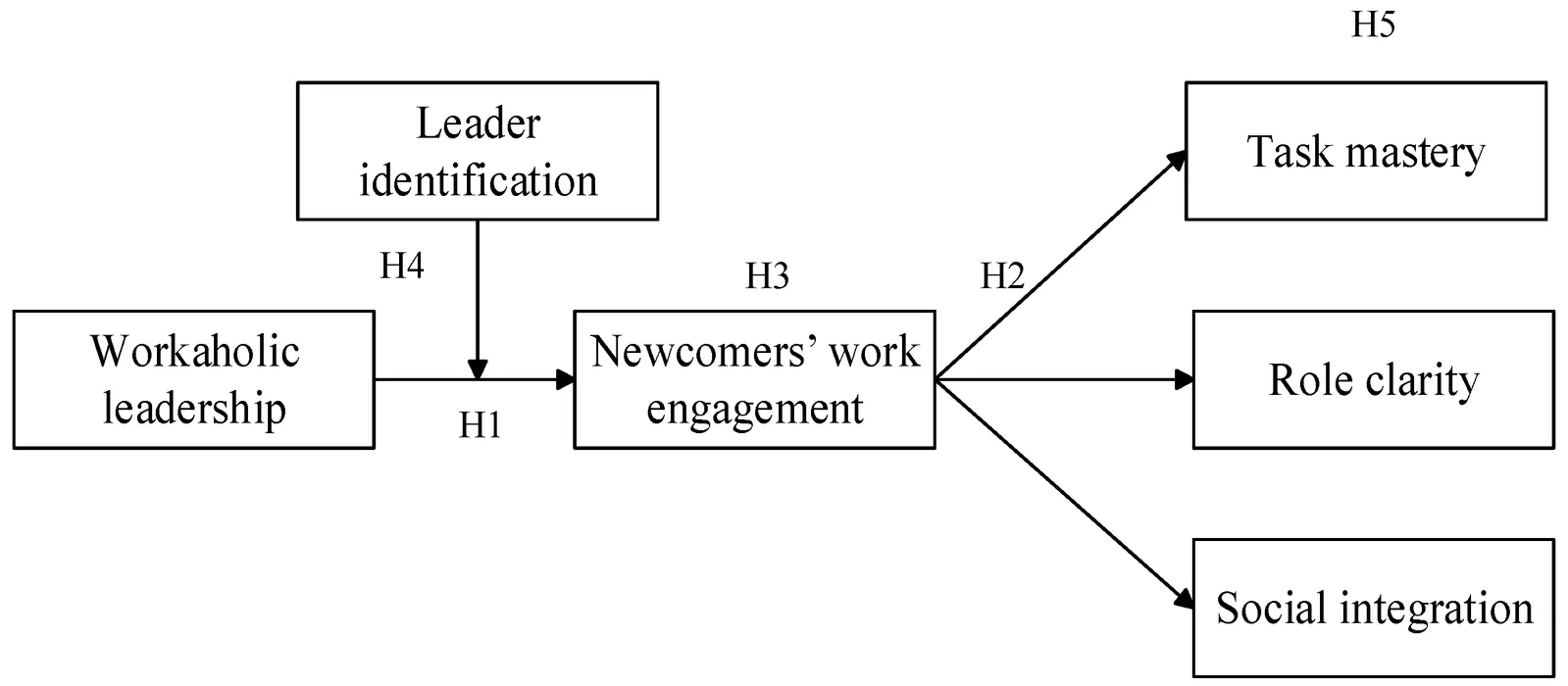
Theoretical model.

To address this research gap, this study thus aims to explore how and when workaholic leadership affects newcomer organizational socialization within the framework of social learning theory. According to social learning theory, individual behavior is shaped not only by external environmental factors but also by observing and imitating role models (Bandura, 1977). Workaholic leaders function as prominent behavioral exemplars at work (Friedman & Lobel, 2003), and this role modeling effect becomes more pronounced in high power distance Confucian cultures, where newcomers tend to emulate leaders’ work norms to speed up organizational adjustment. Accordingly, we posit work engagement, a persistent, enthusiastic work state (Eldor, 2016), as a mediating pathway linking workaholic leadership to newcomer socialization (Ellis et al., 2017). We further introduce leader identification as a boundary condition. Leader identification refers to the degree to which employees incorporate leaders’ traits into their self-perception and follow their behavioral cues (Sluss & Ashforth, 2007; Ashforth et al., 2016). Existing evidence confirms that stronger identification amplifies employees’ behavioral imitation and responsiveness to leader expectations (P. Wang & Rode, 2010). We therefore expect newcomers’ leader identification to moderate both the direct impact of workaholic leadership and work engagement, as well as the indirect path toward socialization outcomes. Figure 1 illustrates the conceptual model for this study.

## 2. Theoretical Background and Research Hypotheses

### 2.1. Workaholic Leadership and Newcomer Work Engagement

Workaholic leadership refers to leaders who exhibit tendencies toward work addiction in the workplace (Clark et al., 2016a). Such leaders are often strongly driven by their work itself and become excessively immersed in work activities, frequently at the expense of other life domains. Research indicates that when leaders display workaholic tendencies, they are more likely to invest substantial effort in their work and demonstrate heightened work motivation, which in turn encourages employees to engage more actively in their tasks, reduces withdrawal behaviors, and enhances organizational citizenship behaviors (Pan, 2018). These effects further contribute to improved employee performance (Friedman & Lobel, 2003) and the cultivation of a positive team atmosphere.

Work engagement is a positive psychological state displayed by newcomers, characterized by both capability and willingness (Demerouti et al., 2010), which reflects their commitment to work. Highly engaged newcomers can effectively invest their physical, cognitive, and emotional energy in their tasks, resulting in increased effort and enhanced focus, which in turn leads to a deeper understanding of their work. This study suggests that workaholic leadership could positively influence newcomer work engagement for the following reasons.

Social learning theory suggests that individuals acquire knowledge and adjust their behaviors by observing and imitating significant others (Bandura, 1977). During organizational socialization, direct supervisors serve as important sources of social information and behavioral cues for newcomers, exerting a substantial influence on their attitudes and behaviors (Manz & Sims, 1981; Taormina, 2008). Prior research has shown that workaholic leadership may generate both positive and negative outcomes. On the one hand, leaders’ high work involvement can convey positive work values and enhance employees’ work motivation and performance (Zhan et al., 2026). On the other hand, the intensive work demands associated with workaholic leadership may contribute to employee fatigue, emotional exhaustion, and silence behavior (Knoll et al., 2025; Qiu et al., 2025; Zhan et al., 2026). However, for newcomers in the early stage of organizational socialization, the primary task is to understand organizational norms and adapt to their new roles (Bauer & Erdogan, 2011; Chong et al., 2021). Given their limited organizational experience, newcomers are more likely to rely on behavioral cues provided by their supervisors to understand organizational expectations and guide their behaviors. Therefore, compared with the potential strain associated with workaholic leadership, newcomers may pay greater attention to the information and role-modeling signals conveyed by leaders’ behaviors. This tendency may be particularly salient in Confucian cultural contexts that emphasize respect for authority and diligence. In such contexts, leaders’ high work involvement is more likely to be perceived as an organizationally endorsed way of working. By observing and interpreting leaders’ workaholic behaviors (Atroszko et al., 2025), newcomers gradually develop an understanding of organizational expectations and adjust their behaviors through observational learning and imitation (Clark et al., 2016b). Therefore, during the early stage of organizational socialization, the social learning effects of workaholic leadership may be more salient than its potential negative consequences, thereby facilitating newcomers’ organizational socialization. Based on the above arguments, we propose the following hypothesis:

**H1.**  *Workaholic leadership is positively related to newcomer work engagement*.

### 2.2. The Mediating Role of Work Engagement

Organizational socialization describes the process by which newcomers learn the knowledge and skills needed for their jobs and gradually settle into formal organizational membership (Louis, 1980). Prior research on organizational socialization has validated three core dimensions (i.e., task mastery, role clarity, and social integration) as standard metrics to evaluate how well newcomers adapt to organizations (Bauer et al., 2025; Zhao et al., 2023), because these three dimensions comprehensively cover practical job competence, cognitive understanding of job roles, and interpersonal inclusion at work. This study thus evaluates newcomer socialization level across these three indicators. In particular, task mastery reflects newcomers’ self-rated ability to complete core work tasks and master necessary job skills (Kammeyer-Mueller & Wanberg, 2003). Role clarity captures the extent to which newcomers understand their job duties, work goals, and relevant implementation approaches. Social integration refers to newcomers’ capacity to develop interpersonal bonds and embed themselves within the organization’s internal social network.

Social learning theory provides a theoretical foundation for understanding how newcomers achieve such adaptive outcomes during organizational entry. From this perspective, newcomer socialization can be viewed as a continuous learning process. Through direct work experience, social interaction, and ongoing feedback, newcomers gradually learn organizational norms, role expectations, and appropriate workplace behaviors, and adjust their attitudes and actions to align with organizational requirements (Bandura, 1977).

Within this social learning process, work engagement serves as a critical pathway that translates leaders’ role modeling into adaptive socialization outcomes. According to social learning theory, individuals develop new behavioral patterns through ongoing experiential learning and behavioral observation (Bandura, 1977). Workaholic leaders consistently demonstrate intensive work focus and persistent work investment, which creates salient behavioral models for newcomers. In Confucian cultural contexts that value diligence and dedication, newcomers are more likely to perceive such leader behaviors as organizationally desirable and worthy of imitation. By observing and modeling their leaders’ high work intensity, newcomers correspondingly increase their own work engagement (Son & Kim, 2021). Higher work engagement further motivates newcomers to proactively devote time and effort to job tasks (Bakker & Demerouti, 2008), allowing them to accumulate task-related knowledge and skills more rapidly and ultimately improve their task mastery.

Meanwhile, social learning theory emphasizes the reciprocal interaction between individuals and their social environments, suggesting that active engagement enables individuals to reshape and refine their contextual understanding (Bandura, 1977). In cultures characterized by high power distance and authority respect (Hofstede, 2001), newcomers are particularly sensitive to role expectations conveyed by leaders and tend to actively seek their feedback to reduce uncertainty. Highly engaged newcomers exhibit greater proactive work tendencies (Crant, 2000), enabling them to acquire role-relevant information from leaders and coworkers through diverse informal and formal channels. As key organizational socialization agents (Taormina, 1997), leaders and coworkers provide newcomers with valuable information regarding task requirements, behavioral norms, and role boundaries (Ostroff & Kozlowski, 1992). Through this ongoing information acquisition, engaged newcomers develop a clearer understanding of their job responsibilities and organizational expectations, thereby improving role clarity.

Finally, work engagement further promotes newcomers’ social integration. Social learning theory highlights that individual behavioral development relies on continuous social participation and contextual interaction, with social environments fundamentally shaping individuals’ behavioral tendencies (Bandura, 1977). During organizational entry, leaders and coworkers constitute the primary social context from which newcomers observe, imitate, and internalize organizational norms (Sluss et al., 2012). In collectivist cultural settings, work engagement further motivates newcomers to participate actively in organizational activities (J. Xu et al., 2019). Engaged newcomers are willing to initiate interpersonal communication, seek social support, and engage in proactive relationship building (Ellis et al., 2017). As such, sustained work participation and frequent interpersonal interaction further accelerate newcomers’ understanding of team norms. Through continuous feedback exchange and relational development, newcomers effectively establish workplace social bonds and gradually integrate into the team, which promotes their social integration within the organization.

Therefore, we propose the following hypotheses:

**H2.**  *Newcomers’ work engagement positively affects (a) task mastery, (b) role clarity, and (c) social integration*.

In sum, workaholic leadership shapes newcomers’ socialization outcomes through a sequential learning process. By demonstrating consistent high work involvement, workaholic leaders provide salient behavioral models for newcomers. Newcomers emulate these behavioral norms and accordingly elevate work engagement, which in turn facilitates task mastery, role clarity, and social integration. Work engagement thus serves as a critical mediating link between workaholic leadership and newcomer socialization. Therefore, we propose the following hypotheses:

**H3.**  *Newcomers’ work engagement mediates the relationship between workaholic leadership and (a) task mastery, (b) role clarity, and (c) social integration*.

### 2.3. The Moderating Effect of Leader Identification

Leader identification reflects employees’ cognitive, affective, and behavioral acceptance and internalization of their leaders (Kark et al., 2003; Sluss & Ashforth, 2007). It represents a psychological state in which employees view their leaders as core representatives of their work group, integrate leaders’ traits, values and goals into their own self-concept, and further align their self-identification with organizational identification (Sluss et al., 2012). For organizational newcomers, leader identification hinges on the value congruence between themselves and their leaders (Graf et al., 2011). Newcomers develop stronger identification with value-consistent leaders, which fosters their sense of collective belonging and accelerates organizational integration (Ellemers et al., 2004).

Social learning theory explains how leader identification shapes newcomers’ behavioral responses. Individuals’ behaviors are guided by cognitive evaluations of environmental cues and role models (Bandura, 2001). In organizational settings, leader identification motivates newcomers to evaluate and reshape their self-concept based on leaders’ behavioral styles and value orientations (Sluss & Ashforth, 2007). The degree of leader identification determines the extent to which newcomers treat their leaders as legitimate learning targets and adjust their own behavioral standards accordingly (Sluss & Ashforth, 2007).

Workaholic leadership features high work dedication and a strong focus on work efficiency. As newcomers generally lack sufficient workplace information and clear role cognition in the early entry stage, they naturally rely on leaders as key behavioral referents to understand workplace norms and adjust their behaviors (Liu et al., 2021; Nasr et al., 2019). More importantly, stronger leader identification enables newcomers to internalize their leaders’ work values and behavioral standards into self-concept (Ashforth et al., 2016). Newcomers with high leader identification are more willing to recognize and emulate leaders’ intensive work patterns, thereby developing more positive work attitudes and higher work engagement. Accordingly, we propose the following hypothesis:

**H4.**  *The higher the degree of leader identification among newcomers, the stronger the positive impact of workaholic leadership on their work engagement*.

### 2.4. Integrated Model: Moderated Mediating Role

Based on the above hypothesis, we propose a moderated mediation model. In this model, newcomers’ identification with their leader may moderate the indirect effects of workaholic leadership on newcomers’ task mastery, role clarity, and social integration through work engagement. Specifically, when newcomers have a higher level of leader identification, the positive impact of workaholic leadership on their work engagement will be strengthened, thereby enhancing the indirect effects of workaholic leadership on newcomers’ task mastery, role clarity, and social integration through work engagement. Conversely, when leader identification is lower, the positive correlation between workaholic leadership and newcomers’ work engagement will be weaker, thereby reducing the indirect effects on task mastery, role clarity, and social integration. Consequently, we propose the following hypothesis:

**H5.**  *Leader identification moderates the indirect effect of workaholic leadership on (a) task mastery, (b) role clarity, and (c) social integration via work engagement. The indirect effect is stronger at higher levels of leader identification and weaker at lower levels of leader identification*.

## 3. Research Methods

### 3.1. Participants and Research Procedures

We conducted data collection at a textile organization in Shandong Province. Before starting the investigation, we explained the survey process in detail to the participants, made a clear commitment to keep their responses strictly confidential, and stated that the collected data and information would be used solely for comprehensive statistical analysis for academic research purposes, not for case analysis or for their performance evaluations. After negotiating with the human resources department, we conducted the survey using paper questionnaires. Each participant was assigned a unique number to match their responses at different stages, and each questionnaire was accompanied by an explanation letter that clearly stated the research objectives and emphasized the protection of participants’ privacy and security again. Participants completed the questionnaire and submitted it directly to the research team.

Given that the effects of leadership behaviors on employee attitudes and behaviors typically emerge gradually through sustained interactions (Sluss & Thompson, 2012), and following the research design of G. Chen and Klimoski (2003) on newcomer performance and organizational socialization, this study implemented a one-month interval between each round of data collection. This interval not only provides temporal separation to reduce common method bias (Podsakoff et al., 2024) and, to some extent, mitigate sample attrition and contextual interference but also allows sufficient time for employees’ work-related perceptions and behavioral changes to form and stabilize (Selig & Little, 2012). In addition, consistent with prior research examining the influence of leadership styles on newcomer organizational socialization (Sluss & Thompson, 2012), this study employed newcomer self-reports to assess perceptions of leadership behaviors. The initial sample consisted of 456 newcomers. At Time 1, newcomers hired within the past year were asked to report their evaluations of workaholic leadership, leader identification, and demographic information. At Time 2, they reported work engagement. At Time 3, they reported their task mastery, role clarity, and social integration. In the final sample, complete data were obtained from 375 newcomers (80.65% of the initial sample). The majority of respondents were male workers (60.3%), held a bachelor’s degree (37.3%), were in non-managerial positions (69.1%), had a tenure of less than one month (52.3%), and were non-fresh graduates (51.5%). The average age of the participants was 27.92 years (*SD* = 3.177), ranging from 22 to 36 years.

### 3.2. Measuring Method

Following the back-translation procedure, survey items were translated from English to Chinese. Unless otherwise specified, all items were measured on a 7-point Likert scale (1 = ‘strongly disagree’, 7 = ‘strongly agree’).

### 3.3. Workaholic Leadership

In this study, workaholic leadership was defined as the immediate supervisors who exhibit workaholic tendencies. Workaholic leadership was measured using the scale developed by Schaufeli et al. (2009), which consists of 10 items. Consistent with prior research, newcomers were asked to evaluate the workaholic behaviors of their direct leaders. The scale captures two dimensions—working excessively and working compulsively—each measured with five items. Sample items include “My direct leader often feels pressed for time” and “I think working hard is very important to my direct leader.” The Cronbach’s alpha for this scale was 0.956.

### 3.4. Leader Identification

Following the research design of X. Wang et al. (2025), leader identification was measured using an adapted version of the organizational identification scale developed by Becker (1992). The scale consisted of five items. A sample item is “I care a great deal about how others view my team leader.” The Cronbach’s alpha for this scale was 0.967.

### 3.5. Work Engagement

Schaufeli et al. (2006) developed a short version of the scale. The scale has nine items and three dimensions: vitality, dedication, and concentration. Representative topics include “I am passionate about work” (vitality), “I like high intensity work” (dedication), and “I often ignore other things while working” (focus). The Cronbach’s alpha for this scale was 0.946.

### 3.6. Task Mastery

We measured task mastery with a five-item scale developed by Chao et al. (1994). A sample item is “I have mastered the necessary skills to complete the task.” The Cronbach’s alpha for this scale was 0.895.

### 3.7. Role Clarity

Six-item scales measuring role clarity were derived from the works of Rizzo et al. (1970). A sample item is “I know my rights at work very well.” The Cronbach’s alpha for this scale was 0.946.

### 3.8. Social Integration

Chao et al. (1994) developed a six-item scale to measure social integration. A sample item is “I believe most of my colleagues in the company like me.” The Cronbach’s alpha for this scale was 0.920.

### 3.9. Control Variables

According to previous studies, age, gender, education level, organizational tenure, work experience, and position were controlled because they have been shown to influence newcomer adaptation (Ellis et al., 2017; Jokisaari & Nurmi, 2009).

### 3.10. Common Method Deviation Test

Given that the data in this study were primarily obtained from self-reported measures, we employed the unmeasured latent method variable (UMLV) approach to address potential concerns regarding common method bias. Model fit comparisons indicated that, relative to the baseline model, the UMLV model (*χ*^2^ = 225.320, *df* = 173, CFI = 0.994, TLI = 0.992, RMSEA = 0.028, SRMR = 0.018) did not demonstrate a statistically significant improvement in model fit (baseline model: *χ*^2^ = 248.917, *df* = 194, CFI = 0.993, TLI = 0.992, RMSEA = 0.027, SRMR = 0.024).

### 3.11. Confirmatory Factor Analysis

In order to ensure the validity of the measurement model, we conducted a series of confirmatory factor analyses (CFA) on six scales in Mplus 8.3 software, including workaholic leadership, leader identification, newcomers’ work engagement, task mastery, role clarity, and social integration. As shown in Table 1, these constructs’ values of standardized factor loadings (ranged from 0.721 to 0.986), CR (ranged from 0.895 to 0.967), and AVE (ranged from 0.631 to 0.856) met the threshold values of 0.50, 0.70, and 0.50, respectively (Fornell & Larcker, 1981; Nunnally & Bernstein, 1994). Thus, these results support each construct’s item reliability, composite reliability, and convergent validity. The square root of the AVE value of each construct is shown in bold on the diagonal of Table 2, which is higher than its correlation with other variables, thus proving qualified discriminant validity.

In addition, we utilized the item parceling approach to reduce the number of parameters in the structural equation model analysis and maintain a reasonable degree of freedom (Kline, 2023). Specifically, for the workaholic leadership and the work engagement, three parcels were formed by combining items from each facet, with each parcel containing three or four items (Clark et al., 2020); for the role clarity and the social integration, three parcels were formed by random allocation to maximize the internal consistency of the packaged items. Then, we conducted CFAs to compare the hypothetical six-factor model against alternative models with different combinations of constructs. The results showed that the six-factor model [*χ*^2^ (194) = 248.962, *p* < 0.010, CFI = 0.993, RMSEA = 0.027, SRMR = 0.024] is better than other models for data fitting.

### 3.12. Descriptive Statistics

Table 2 summarizes the studied variables’ means, standard deviations, and correlations. As mentioned above, workaholic leadership was positively correlated with newcomers’ work engagement (*r* = 0.527, *p* < 0.010). Work engagement was positively correlated with their task mastery (*r* = 0.449, *p* < 0.010), role clarity (*r* = 0.198, *p* < 0.010), and social integration (*r* = 0.151, *p* < 0.010). The results of the statistical test of correlations between these variables provide preliminary evidence to support the proposed hypotheses.

### 3.13. Hypothesis Testing

We utilized Mplus 8.3 to test our hypotheses. For the mediating effect, as shown in Table 3, when controlling for all control variables, workaholic leadership was positively related to newcomers’ work engagement (*B* = 0.464, *SE* = 0.037, *p* < 0.001), supporting H1. At the same time, newcomers’ work engagement was positively associated with task mastery (*B* = 0.470, *SE* = 0.057, *p* < 0.001), role clarity (*B* = 0.224, *SE* = 0.059, *p* < 0.001), and social integration (*B* = 0.182, *SE* = 0.064, *p* < 0.001), supporting H2. In addition, to test the mediating effect of newcomers’ work engagement, we bootstrapped 5000 samples to construct a 95% bias corrected confidence interval (CI). Results indicated that workaholic leadership was significantly and indirectly related to task mastery (indirect effect = 0.257, *SE* = 0.037, *p* < 0.001, 95% CI = [0.184, 0.330]), role clarity (indirect effect = 0.122, *SE* = 0.037, *p* < 0.010, 95% CI = [0.056, 0.188]), and social integration (indirect effect = 0.094, *SE* = 0.034, *p* < 0.010, 95% CI = [0.027, 0.162]) through newcomers’ work engagement, with 95% CI excluding zero. However, after controlling for newcomers’ work engagement, the direct effect of workaholic leadership on task mastery remained significant, whereas its direct effects on role clarity and social integration were not significant. These results indicate that newcomers’ work engagement mediates the relationships between workaholic leadership and task mastery, role clarity, and social integration, thus supporting H3.

**Figure 2:**
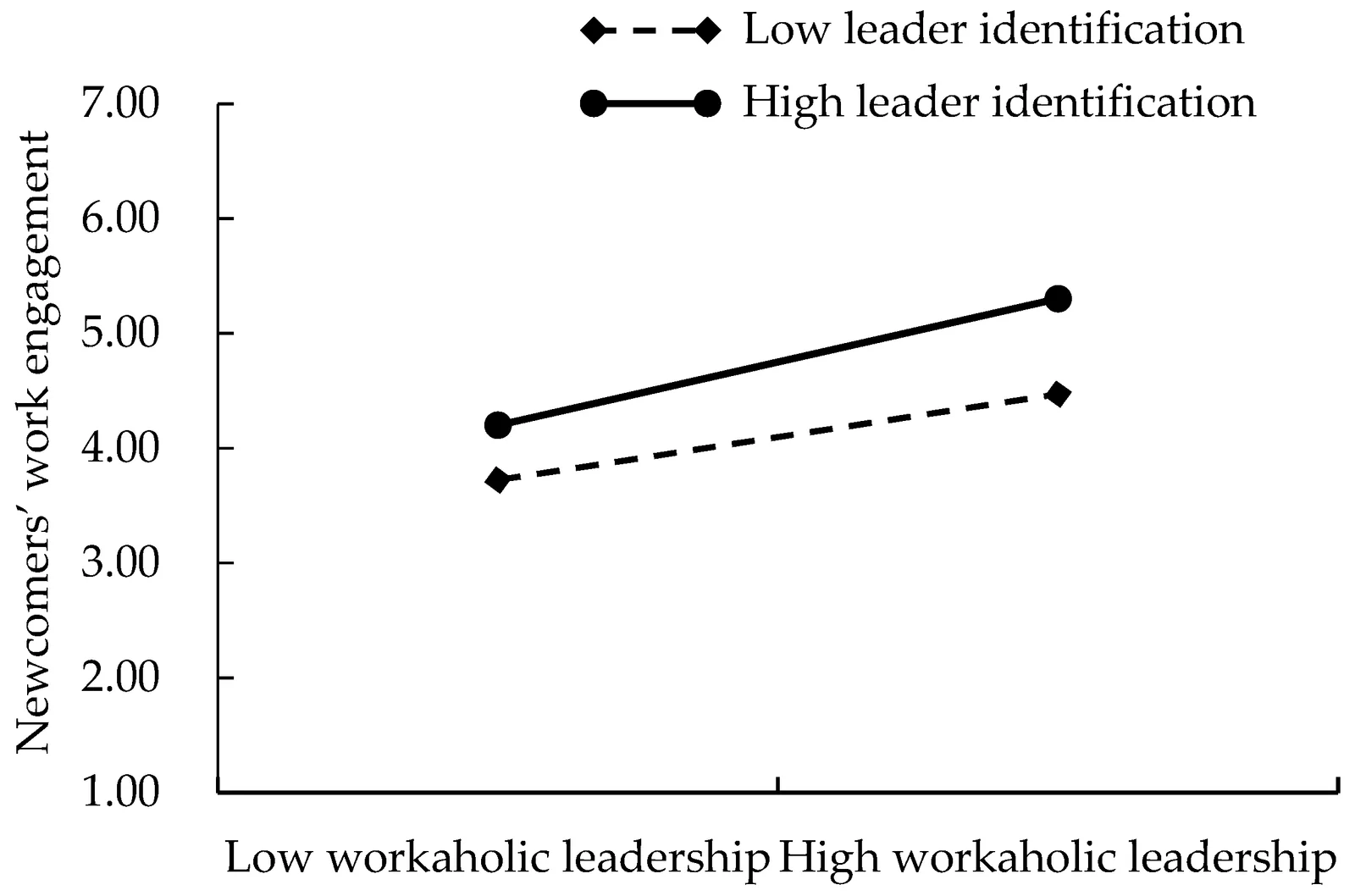
The moderating role of leader identification in the relationship between workaholic leadership and newcomers’ work engagement.

To test H4, we used Mplus 8.3 to examine the interaction effects. The results presented in Table 3 indicated a significant interaction effect between workaholic leadership and leader identification in relation to newcomer work engagement (*B* = 0.090, *SE* = 0.024, *p* < 0.001). To further clarify these interactions, we performed simple slope analyses. The results showed that for newcomers perceiving high levels of leader identification (M + 1 SD), workaholic leadership was strongly associated with newcomer work engagement (*B* = 0.554, *SE* = 0.043, 95% CI = [0.471, 0.637]), For those perceiving low levels of leader identification (M − 1 SD), workaholic leadership was still significantly associated with newcomer work engagement (*B* = 0.374, *SE* = 0.045, 95% CI = [0.288, 0.467]), but this positive association was weaker (see Figure 2). This indicated that leader identification enhanced the positive effect of workaholic leadership on newcomers’ work engagement, supporting H4.

As shown in Table 4, the analysis of the moderated mediation models revealed the indirect effects of workaholic leadership on newcomers’ task mastery, role clarity, and social integration, moderated by leader identification. Specifically, when leader identification was high (M + 1 SD), workaholic leadership exhibited significant indirect effects on task mastery (*B* = 0.261, *SE* = 0.039, 95% CI = [0.189 0.330]), role clarity (*B* = 0.124, *SE* = 0.034, 95% CI = [0.059, 0.185]), and social integration (*B* = 0.101, *SE* = 0.036, 95% CI = [0.034, 0.164]) through work engagement. Conversely, when leader identification was low (M − 1 SD), workaholic leadership exhibited weak indirect effects on task mastery (B = 0.176, SE = 0.032, 95% CI = [0.119, 0.234]), role clarity (*B* = 0.084, *SE* = 0.025, 95% CI = [0.039, 0.129]), and social integration (*B* = 0.068, *SE* = 0.027, 95% CI = [0.023, 0.117]) through newcomers’ work engagement. Moreover, the differences between high and low leader identification conditions were significant for task mastery (*B* = 0.085, *p* < 0.010, 95% CI = [0.041, 0.138]), role clarity (*B* = 0.040, *p* < 0.010, 95% CI = [0.016, 0.077]), and social integration (*B* = 0.033, *p* < 0.010, 95% CI = [0.012, 0.067]), supporting H5.

## 4. Discussion

This study found that workaholic leadership can facilitate newcomers’ task mastery, role clarity, and social integration by enhancing their work engagement. This finding suggests that the effects of workaholic leadership are not limited to resource-depletion mechanisms; rather, its outcomes may vary depending on employees’ developmental stage and the organizational context. Previous research has produced mixed findings regarding the consequences of workaholic leadership. Research grounded in the Conservation of Resources theory suggests that workaholic leaders often convey high work demands and persistent work pressure to employees, which may deplete employees’ resources and subsequently lead to negative outcomes such as psychological distress, emotional exhaustion, and work fatigue (Dong & Li, 2024; Qiu et al., 2025). At the same time, other studies have shown that the high levels of dedication and hard-working behaviors displayed by workaholic leaders can promote employees’ positive work behaviors and job performance (She et al., 2021; She et al., 2024). Together, these findings indicate that the effects of workaholic leadership are complex and highly contingent upon contextual conditions.

The present study focuses on newcomers in the early stage of organizational socialization. Organizational socialization research suggests that newcomers typically experience considerable uncertainty, information deficits, and role ambiguity upon entering an organization. Their primary tasks involve learning job requirements, understanding organizational norms, and developing a sense of organizational membership (Louis, 1980; Bauer et al., 2007; Bauer et al., 2025). During this process, leaders serve as a critical source of work-related information and organizational expectations (Taormina, 2008; A. S. Lee & Jacobs, 2024). Because newcomers lack sufficient organizational experience and established behavioral reference points, they tend to rely more heavily on social information from their environment to develop an understanding of organizational norms and behavioral standards (Ostroff & Kozlowski, 1992; Nifadkar, 2020). Consequently, leadership behaviors play a particularly important role in transmitting information and providing behavioral guidance during newcomers’ organizational socialization.

According to Social Learning Theory, individuals acquire new cognitive frameworks and behavioral patterns by observing and modeling the behaviors of significant others (Bandura, 1977, 2001). Given their formal authority and control over resources, leaders’ behaviors are highly visible and possess substantial role-model value (Manz & Sims, 1981). The high levels of dedication and persistent effort exhibited by workaholic leaders signal the behavioral standards that are valued within the organization. Through observational learning, newcomers transform these behavioral cues into their own work attitudes and behavioral tendencies, thereby exhibiting higher levels of work engagement. Elevated work engagement, in turn, enables newcomers to invest greater cognitive, emotional, and behavioral resources in their work and adaptation processes, ultimately facilitating task mastery, role clarity, and social integration. Therefore, work engagement serves as an important mechanism through which workaholic leadership influences newcomers’ organizational socialization.

Furthermore, this study found that leader identification strengthens the positive relationship between workaholic leadership and newcomers’ work engagement. Leader identification reflects the degree of psychological connection between employees and their leaders, as well as the extent to which employees incorporate the leader into their self-concept (Sluss & Ashforth, 2007). When leader identification is high, newcomers are more likely to accept the values and behavioral norms conveyed by their leaders and to regard them as important behavioral role models (Ashforth et al., 2016). Under such conditions, the high-engagement behaviors demonstrated by workaholic leaders are more readily internalized as personal behavioral standards, thereby enhancing newcomers’ work engagement. In contrast, when leader identification is low, the informational and role-modeling functions of leadership behaviors become less effective, weakening the positive influence of workaholic leadership on work engagement. This finding highlights leader identification as an important boundary condition shaping the effectiveness of workaholic leadership.

Overall, the findings suggest that, during the early stage of organizational socialization, workaholic leadership can promote newcomers’ organizational socialization by enhancing their work engagement. Leader identification further strengthens this process.

### 4.1. Theoretical Implications

Our study extends research on newcomers’ organizational socialization from a social learning theory perspective. Although prior research has established the important role of leaders in newcomers’ organizational socialization and examined the effects of various leadership styles, most studies have focused on leaders’ influence through organizational support, task guidance, or empowerment (Dai & Fang, 2023; Harris et al., 2014), leaving insufficient attention to how workaholic leaders influence newcomers’ socialization through daily behavioral demonstration. According to social learning theory, behaviors that are repeatedly enacted and highly observable are more likely to be noticed and imitated (Bandura, 1977). As newcomers are in the organizational adaptation stage, they are particularly sensitive to social cues and leaders’ behaviors, and leaders’ work engagement behaviors serve as salient behavioral models that shape newcomers’ socialization learning processes. Based on this perspective, the present study elucidates a unique pathway through which workaholic leadership influences newcomers’ organizational socialization via behavioral modeling, thereby complementing existing behavioral explanations of leadership effects on newcomer socialization and further extending research on the antecedents and mechanisms of organizational socialization (Saks & Gruman, 2014).

Second, although prior research has demonstrated that newcomer organizational socialization contributes to enhancing their work engagement (Islam et al., 2025), empirical research examining how work engagement, in turn, influences newcomers’ organizational socialization processes remains relatively limited. From the perspective of social learning theory, newcomer organizational socialization is not a static outcome, but rather a dynamic process in which individuals continuously learn and understand organizational norms and role requirements during the course of ongoing adaptation (Bauer et al., 2025). Through persistent behavioral demonstration, workaholic leadership reinforces the necessity of high work involvement, thereby encouraging newcomers to devote greater effort to task performance and role enactment. As newcomers’ work engagement increases, they acquire more direct learning experiences, which deepen their understanding of organizational norms and role requirements and, in turn, accelerate their organizational socialization process. Based on this mechanism, the present study identifies the mediating role of work engagement in the relationship between workaholic leadership and newcomers’ organizational socialization, thereby extending the process-based explanation of work engagement within the organizational socialization literature and further responding to calls for deeper investigation into the dynamic process of newcomer organizational socialization.

Third, grounded in social learning theory, our study introduces leader identification as a critical boundary condition. Social learning theory suggests that observation and imitation of role models are selective processes, and the attractiveness and degree of identification with the model determine whether learning occurs and its intensity (Bandura, 1977). Newcomers do not merely passively accept organizational norms; rather, they engage in meaning construction and behavioral choice through interactions with organizational members (Bauer et al., 2025). Leader identification reflects whether employees regard their leaders as important behavioral referents (Sluss & Ashforth, 2007). For newcomers, a high level of leader identification means that they are more likely to perceive the intensive work patterns of workaholic leaders as a role model, thereby strengthening their attention to and imitation of such patterns and enhancing their work engagement. In contrast, lower levels of leader identification weaken this social learning process. The findings indicate that leader identification strengthens the effect of workaholic leadership on newcomer work engagement, thereby clarifying a key psychological prerequisite for the occurrence of social learning and further responding to calls for greater attention to how the effects of workaholic leadership may vary across employee individual differences (Zhan et al., 2026).

### 4.2. Practical Implications

Our research also provides several practical implications for organizations. First, this study emphasizes the benefits of workaholic leadership for organizations. Consistent with previous research demonstrating a positive relationship between workaholic leadership and employees’ work-related behaviors (Zeng & Liu, 2022). Our findings further indicate that workaholic leadership can effectively facilitate newcomer organizational socialization through the mediating role of work engagement. Compared with Western organizational contexts characterized by low power distance and strong individualism, Chinese mainstream culture places greater emphasis on hierarchical order, collective relationships, and long-term orientation (Hofstede, 2001). Consequently, newcomers are more likely to perceive leaders’ high work involvement as an organizationally endorsed behavioral exemplar. However, workaholic behaviors may also produce negative outcomes, such as increased turnover intentions and heightened psychological strain among newcomers. Therefore, organizations should appropriately recognize and actively guide the high standards, strong sense of responsibility, and goal-oriented characteristics associated with workaholic leadership, and, through workload management and psychological support programs, help leaders maintain high performance expectations while also attending to newcomers’ mental health and long-term development, thereby promoting their integration into the organization.

Second, our findings further emphasize that newcomer work engagement can effectively facilitate their organizational socialization process. Within Confucian cultural contexts that emphasize collectivism, newcomers exhibit higher levels of work engagement, which is reflected not only in active participation in work tasks and proactive adaptation to organizational norms and role expectations but also in using work engagement as a means to gain leader recognition and establish a sense of organizational belonging. Therefore, leaders can adopt various strategies to encourage newcomers to actively engage in their work, helping them better understand their task roles and integrate more effectively into the organization. For example, leaders may serve as role models by demonstrating dedication and enthusiasm at work, thereby providing newcomers with positive behavioral examples to follow. Given that newcomers’ work attitudes are more malleable, such modeling can inspire them to invest greater effort and energy into their tasks (B. Wang et al., 2025), which in turn enables them to perform more efficiently and experience greater satisfaction and achievement (Islam et al., 2025). This process not only strengthens newcomers’ sense of identification and belonging to the organization but also contributes to a smoother and more successful socialization process (Bauer et al., 2025).

Third, our results demonstrate that newcomers’ identification with their leaders plays a crucial role in the influence of workaholic leadership. Because leader identification can strengthen the positive effects of leadership behaviors on employees (Yang et al., 2025), organizations should pay attention to the alignment of values between newcomers and their leaders (Graf et al., 2011) to enhance newcomers’ identification with their leaders. Within Confucian cultural contexts that emphasize “learning from superiors” and the role-modeling effect of exemplars, leaders can further reinforce this value alignment by sharing personal experiences, providing developmental guidance, and demonstrating passion and responsibility in their work. These behaviors can help newcomers understand their leaders’ work styles and values, reinforcing value congruence and identification, which in turn enhances leaders’ influence (Ashforth et al., 2016), promotes newcomer engagement, and facilitates their integration into the organization.

### 4.3. Limitations and Future Research

Although our study explored the impact of workaholic leadership on newcomers’ organizational socialization, it inevitably has several limitations. First, the sample of this study was drawn from a single apparel and textile enterprise in Shandong Province, China, which presents clear regional and industry-specific limitations and is also strongly influenced by the Chinese cultural context. Within Confucian cultural contexts, leaders’ influence tends to be further strengthened (Lin & Sun, 2018). As a result, the positive effects of workaholic leadership may be amplified, thereby limiting the generalizability of the findings to other cultural or organizational contexts. Future research could validate the present findings using multi-organizational samples across industries, regions, and cultural contexts to enhance the external validity and explanatory power of the conclusions. Second, although our data were collected from newcomers at three points in time, we cannot draw causal conclusions from such a time-lagged, multi-source investigation. For example, newcomers who are better socialized may be more inclined to actively engage in their work. Therefore, we encourage future research to adopt experimental designs to better identify causality. Second, this study only examines the positive effect of workaholic leadership on newcomers’ organizational socialization through newcomers’ work engagement, without incorporating other potential mediating variables that may influence the socialization process, such as work stress and work-related well-being. Prior research indicates that workaholic leaders emphasize goal attainment, impose high work demands on newcomers, and tend to exhibit relatively low levels of moral concern. As a result, newcomers may experience heightened work stress (Zeng & Liu, 2022), reduced work well-being (Clark et al., 2016b), and strengthened silence behavior (Taris & de Jonge, 2024), which may crowd out the time and psychological resources available for role learning and organizational adaptation. These findings suggest that the influence of workaholic leadership on newcomers’ organizational socialization is inherently dual-sided. However, the present study primarily focuses on its positive effects and does not sufficiently examine the potential risks associated with employee well-being, burnout, and long-term organizational sustainability, leaving room for further normative reflection. Future research may adopt an integrative framework to systematically examine the roles of these negative mechanisms, thereby providing a more comprehensive understanding of the complex effects of workaholic leadership on newcomers’ organizational socialization. Therefore, we recommend that scholars continue to explore the roles of these variables to enrich the research. Third, our study focused on newcomers’ identification with leaders; future research could also consider other behavioral characteristics of newcomers. For example, previous studies have shown that career identity motivates newcomers to align more with their job roles, thereby increasing their willingness to invest time and energy in their work and enhancing their work engagement (Wu et al., 2022). Therefore, future researchers may also consider other boundary conditions (e.g., career identity) when discussing the impact of workaholic leadership on newcomers’ work engagement.

## Figures and Tables

**Table 1:** Confirmatory factor analysis for each construct.

Constructs	Items	SFL	CR	AVE
Workaholic leadership	Workaholic leadership 1	0.832	0.947	0.687
	Workaholic leadership 2	0.859		
	Workaholic leadership 3	0.827		
	Workaholic leadership 4	0.846		
	Workaholic leadership 5	0.837		
	Workaholic leadership 6	0.826		
	Workaholic leadership 7	0.833		
	Workaholic leadership 8	0.823		
	Workaholic leadership 9	0.812		
	Workaholic leadership 10	0.789		
Leader identification	Leader identification 1	0.931	0.967	0.856
	Leader identification 2	0.917		
	Leader identification 3	0.922		
	Leader identification 4	0.929		
	Leader identification 5	0.927		
Work engagement	Work engagement 1	0.766	0.947	0.666
	Work engagement 2	0.776		
	Work engagement 3	0.858		
	Work engagement 4	0.844		
	Work engagement 5	0.800		
	Work engagement 6	0.764		
	Work engagement 7	0.839		
	Work engagement 8	0.933		
	Work engagement 9	0.748		
Task mastery	Task mastery 1	0.774	0.895	0.631
	Task mastery 2	0.817		
	Task mastery 3	0.813		
	Task mastery 4	0.792		
	Task mastery 5	0.776		
Role clarity	Role clarity 1	0.818	0.953	0.773
	Role clarity 2	0.866		
	Role clarity 3	0.846		
	Role clarity 4	0.933		
	Role clarity 5	0.986		
	Role clarity 6	0.812		
Social integration	Social integration 1	0.799	0.921	0.663
	Social integration 2	0.721		
	Social integration 3	0.807		
	Social integration 4	0.798		
	Social integration 5	0.803		
	Social integration 6	0.942		

Notes. SFL = standardized factor loading, CR = composite reliability, AVE = average variance extracted.

**Table 2:** Means, standard deviations, and correlations of variables.

Variables	M	SD	1	2	3	4	5	6	7	8	9	10	11	12
1. Age	27.92	3.177												
2. Gender	0.60	0.490	−0.008											
3. Education	2.36	0.948	0.391 **	0.125 *										
4. Tenure (months)	1.32	1.961	0.321 **	−0.082	0.011									
5. Position	1.69	0.463	−0.079	−0.084	−0.075	0.021								
6. Experience	1.51	0.500	0.539 **	−0.014	−0.245 **	0.208 **	−0.027							
7. Workaholic leadership	4.615	1.318	−0.040	−0.006	−0.012	−0.056	−0.051	0.043	0.829					
8. Leader identification	4.603	1.842	−0.014	−0.008	−0.049	−0.051	−0.133 *	−0.017	0.186 **	0.925				
9. Work engagement	4.465	1.241	0.016	−0.002	−0.058	−0.024	−0.062	0.065	0.527 **	0.533 **	0.816			
10. Task mastery	4.539	1.202	−0.002	−0.080	−0.043	0.044	−0.020	0.018	0.188 **	0.186 **	0.449 **	0.794		
11. Role clarity	4.764	1.207	0.002	0.058	0.045	0.067	−0.013	−0.029	0.071	0.182 **	0.182 **	0.182 **	0.879	
12. Social integration	4.452	1.272	−0.044	0.033	0.019	−0.001	0.034	−0.120 *	0.052	0.200 **	0.151 **	0.187 **	0.297 **	0.814

Notes. *N* = 375. Gender was coded as 1 = males and 2 = females. Education was coded as 1 = associate degree or below, 2 = bachelor’s degree, 3 = master’s degree, and 4 = doctoral degree. * *p* < 0.050, ** *p* < 0.010. The diagonal represents the square root of AVE.

**Table 3:** Results of path analysis.

Variables	Work Engagement			Task Mastery			Role Clarity			Social Integration		
	B	SE	95% CI	B	SE	95% CI	B	SE	95% CI	B	SE	95% CI
Age	0.019	0.022	[−0.024, 0.062]	−0.010	0.029	[−0.066, 0.047]	−0.017	0.033	[−0.085, 0.046]	0.023	0.038	[−0.049, 0.099]
Gender	0.035	0.093	[−0.148, 0.220]	−0.186	0.119	[−0.417, 0.047]	0.142	0.128	[−0.114, 0.339]	0.107	0.138	[−0.166, 0.370]
Education	−0.054	0.062	[−0.176, 0.066]	0.001	0.083	[−0.158, 0.168]	0.080	0.093	[−0.095, 0.275]	−0.051	0.102	[−0.256, 0.145]
Tenure (months)	0.002	0.025	[−0.048, 0.049]	0.035	0.030	[−0.023, 0.094]	0.057	0.032	[−0.007, 0.119]	0.014	0.038	[−0.058, 0.089]
Position	0.048	0.102	[−0.154, 0.247]	−0.008	0.117	[−0.232, 0.228]	0.006	0.131	[−0.240, 0.273]	0.121	0.135	[−0.135, 0.391]
Experience	0.072	0.136	[−0.195, 0.338]	−0.021	0.157	[−0.333, 0.285]	−0.049	0.178	[−0.400, 0.306]	−0.442 *	0.196	[−0.827, −0.066]
Workaholic leadership	0.464 ***	0.037	[0.389, 0.534]	−0.060	0.055	[−0.171, 0.045]	−0.041	0.055	[−0.151, 0.067]	−0.028	0.065	[−0.155, 0.096]
Work engagement				0.470 ***	0.057	[0.359, 0.583]	0.224 ***	0.059	[0.108, 0.345]	0.182 **	0.064	[0.062, 0.312]
Leader identification	0.328 ***	0.026	[0.277, 0.378]									
Workaholic leadership × Leader identification	0.090 ***	0.024	[0.042, 0.136]									

Note: *N* = 375. CI = confidence interval. * *p* < 0.050, ** *p* < 0.010, *** *p* < 0.001.

**Table 4:** Results for moderated mediation effects.

Paths	Moderating Variable	B	SE	95% CI
Workaholic leadership→ Work engagement → Task mastery	Low leader identification	0.176 ***	0.032	[0.119, 0.234]
	High leader identification	0.261 ***	0.039	[0.189, 0.330]
	Difference	0.085 **	0.024	[0.041, 0.138]
Workaholic leadership→ Work engagement → Role clarity	Low leader identification	0.084 **	0.025	[0.039, 0.129]
	High leader identification	0.124 ***	0.034	[0.059, 0.185]
	Difference	0.040 **	0.015	[0.016, 0.077]
Workaholic leadership → Work engagement → Social integration	Low leader identification	0.068 *	0.027	[0.023, 0.117]
	High leader identification	0.101 **	0.036	[0.034, 0.164]
	Difference	0.033 **	0.014	[0.012, 0.067]

Note: *N* = 375. CI = confidence interval. * *p* < 0.050, ** *p* < 0.010, *** *p* < 0.001.

## Data Availability

The data supporting the findings of this study are available from the corresponding author upon reasonable request.
